# Identification and molecular epidemiology of methicillin resistant *Staphylococcus pseudintermedius* strains isolated from canine clinical samples in Argentina

**DOI:** 10.1186/s12917-019-1990-x

**Published:** 2019-07-27

**Authors:** Paula Gagetti, Alice R. Wattam, Gabriela Giacoboni, Adriana De Paulis, Eugenia Bertona, Alejandra Corso, Adriana E. Rosato

**Affiliations:** 1 Servicio Antimicrobianos INEI-ANLIS “Dr. Carlos G. Malbran” , CABA, Argentina; 2 Biocomplexity Institute, University of Virginia, Virginia, USA; 3 Departamento de Microbiologia, Facultad de Ciencias Veterinarias-UNLP, La Plata, Argentina; 4 Departamento Microbiología, Instituto de Investigaciones Médicas Alfredo Lanari-UBA, CABA, Argentina; 5 Department of Pathology and Genomic Medicine, Houston Methodist Research Institute, Houston, TX USA

**Keywords:** MRSP, Antibiotic resistance, Zoonosis, Small animals

## Abstract

**Background:**

*Staphylococcus pseudintermedius* is the leading cause of pyoderma in dogs and the frequent use of antimicrobial treatment is associated to the development of resistance to nearly all classes of antibiotics. Despite *S. pseudintermedius* significance, our understanding of the molecular mechanism of β-lactam resistance and its genetic diversity remains limited. We aimed to: *i*) determine the phenotypic resistance profile of methicillin resistant *Staphylococcus pseudintermedius* (MRSP) isolated from infected dogs in three different veterinary hospitals in Buenos Aires, Argentina; *ii*) identify the SCC*mec* elements and resistance genes; and *iii*) analyze the clonal relationship between isolates and in regard of dominant lineages found in the world.

**Results:**

In addition to the differential levels of β-lactam resistance, MRSP isolates (*n* = 10) showed resistance to 5–6 families of antibiotics, and were therefore categorized as multidrug-resistant. All the isolates were variant of SCC*mec* V homologous to *S. aureus*; additional SCC*mec*Finder analysis classified five of the genomes as SCC*mec* type V (5C2&5) with *mecA* (encodes for PBP2a)*, mecRI* and *mecI* and all the genes closely related to the reference SCC*mec* type V *S. aureus* TSGH17 strain. In the remaining five strains, *mecA* was present, although other genes associated with SCC*mec* V including *mecR1* and *mecI* were missing. PBP2a was inducible in low level resistance strains (MRSP 8151), and constitutively expressed in MRSP 8150, suggesting different *mecA* regulatory mechanisms. MRSP isolates showed significant genetic diversity: eight PFGE clonal types and six multilocus-sequence typing (MLST) sequence types (STs) (339, 649, 919, 920, 921 and 922), including four new STs genetically distinct from STs reported in other geographic areas. Comparative genomics and phylogenetic analyses of the MRSP showed a correlation between the genetic content and the phenotypes, and established the genetic relationship between the isolates.

**Conclusions:**

MRSP could be a threat to animal health due to it concerning level of antimicrobial resistance. Our study highlights genetic and epidemiological aspects of multidrug-resistant MRSP strains from Argentina showing high degree of correlation between the resistance genes and the phenotype of the isolates and, furthermore, they appeared evolutionary closer to major worldwide reported ST68 and ST71.

**Electronic supplementary material:**

The online version of this article (10.1186/s12917-019-1990-x) contains supplementary material, which is available to authorized users.

## Background

*S. pseudintermedius* is an important pathogen in dogs and cats and is sporadically associated with human infections [1]. Over the past decade, methicillin resistant *S. pseudintermedius* (MRSP) has emerged in different parts of the world and has become one of the most important bacterial pathogens in small-animal-veterinary-medicine [2, 3]. Based on data from the World Health Organization (WHO) and the United Nations in 2016, antimicrobial resistance in humans, like in companion animals, represents a problem for public health.

The β-lactam resistance of MRSP is due to penicillin-binding protein 2a (PBP2a), a protein encoded by the methicillin resistant gene *mecA*. This gene is known to reside in a mobile genetic element, a staphylococcal cassette chromosome designated SCC*mec* that contains the *mec* gene complex, *mecA* and some additional genes, and the cassette chromosome recombinase (*ccr*) gene complex, which is responsible for insertion of the SCC*mec* cassette into the core genome. So far as many as thirteen different structural types of SCC*mec* have been described in *S. aureus* based on the different combinations of class of *mec* complexes according to the presence/absence of regulatory genes and insertion sequences, and *ccr* allotypes (*ccrAB* and *ccrC*) [4]. Eleven main types, subtypes, and variants have already been described in the database of the International Working Group on the Staphylococcal Cassette Chromosome (IWG-SCC) [5]. Some *S. aureus* and coagulase-negative *Staphylococcus* isolates carry a *mecA* homolog, *mecC*, which has been recently reported carried by SCC*mec* XI [6, 7]. The classification of SCC*mec* elements is complex, given that there are composite cassettes and pseudo-SCC*mec* elements that do not harbor *ccr* genes [8]. While in *S. aureus* the structure of SCC*mec* elements has been shown to be relatively stable, in MRSP the SCC*mec* elements showed high genetic diversity [4].

The cefoxitin disk is considered as the main method for methicillin resistance detection in *S. aureus*, nevertheless this is not an accurate method of screening for methicillin resistance in *S. pseudintermedius* [9, 10], that must be detected with the oxacillin disk.

In Argentina, previous studies have revealed a prevalence of methicillin resistance between 10 and 30% of *S. pseudintermedius* clinical isolates obtained from dogs [11, 12].

Several dominant MRSP lineages have been identified in the world, including ST45, ST68 and ST71 [8], but the molecular epidemiology of MRSP clones circulating in Argentina has not been examined.

The aims of this study were to determine the phenotypic resistance profile of MRSP, to identify the SCC*mec* elements and resistance genes, to analyze the clonal relationship between isolates, and to compare these isolates with the dominant lineages found globally.

## Results

### Antibiotic resistance profiles

All the *S. pseudintermedius* isolates were considered resistant to oxacillin based on the recently revised Clinical and Laboratory Standards Institute (CLSI) breakpoints of *S. pseudintermedius*, the presence of *mecA* gene and a SCC*mec* element. We identified from the total of 10 MRSP strains two distinct groups with different expression of β-lactam resistance. Six of the ten isolates displayed low-level of oxacillin resistance with minimal inhibitory concentration (MIC) values in the range of 0.5 to 2 mg/L despite being *mecA* positive, and the rest (4 of 10 isolates) exhibited high-level resistant (≥8 mg/L) (Table 1). All the strains were resistant to oxacillin, penicillin, streptomycin and kanamycin. In addition to the β-lactam resistance gene *mecA*, all the isolates contained the β-lactamase gene *blaZ*, the kanamycin and neomycin phosphotransferase gene *aph (3′)-III* and the streptomycin adenylyl-nucleotidyltransferase gene *ant(6)-Ia*. Resistance to macrolides, lincosamides, and streptogramins-B seen in eight isolates was due to the methylase gene *ermB,* and all of them displayed constitutive resistance to clindamycin. The nine trimethoprim/sulfamethoxazole-resistant isolates contained the dihydrofolate reductase gene *dfrG,* and the three tetracycline resistant isolates carried the tetracycline and minocycline resistance gene *tetM*. Only one strain was resistant to gentamicin, and it had the *aac(6′)-Ie–aph(2′)-Ia* gene. Table 2 summarizes the genes associated with resistance and the antimicrobial resistance phenotype displayed by the isolates.

**Table 1:**
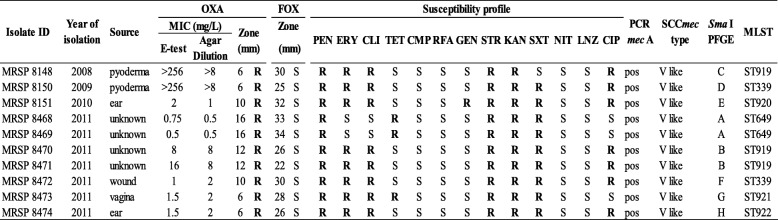
Phenotypic and genotypic characteristics of the MRSP strains

*OXA* oxacillin, *FOX* cefoxitin, *PEN* penicillin, *ERY* erythromycin, *CLI* clindamycin, *TET* tetracycline, *CMP* chloramphenicol, *RFA* rifampicin, *GEN* gentamicin, *STR* streptomycin, *KAN* kanamycin, *SXT* trimethoprim-sulfamethoxazole, *NIT* nitrofurantoin, *LNZ* linezolid and *CIP* ciprofloxacin, *S* susceptible, *R* resistant.

**Table 2:** Resistance Genes and Antimicrobial Resistance Phenotypes of the MRSP strains

No. of Isolates	Resistance Genes	Antimicrobial Resistance Phenotype
5	*mecA*, *blaZ*, *ermB, dfrG, aph*(3`)-III, *ant*(6)-Ia	OXA, PEN, ERY, CLI, STR, KAN, SXT, CIP*
2	*mecA*, *blaZ*, *tetM*, *dfrG*, *aph*(3`)-III, *ant*(6)-Ia	OXA, PEN, TET, STR, KAN, SXT
1	*mecA*, *blaZ*, *ermB, dfrG, aph*(3`)-III, *ant*(6)-Ia, *aac*(6′)-*aph(*2″)	OXA, PEN, ERY, CLI, GEN, STR, KAN, SXT, CIP*
1	*mecA*, *blaZ*, *ermB, tetM, dfrG, aph*(3`)-III, *ant*(6)-Ia	OXA, PEN, ERY, CLI, TET, STR, KAN, SXT
1	*mecA, blaZ, ermB, aph*(3`)-III, *ant*(6)-Ia	OXA, PEN, ERY, CLI, STR, KAN, CIP*

* The amino acid substitutions related to the CIP resistance are shown in Table 3

In addition, mutations in the quinolone resistance-determining region (QRDR) of the topoisomerase genes of the seven ciprofloxacin-resistant isolates were found, including amino acid substitution S84 L in the topoisomerase GyrA and S80I in GrlA. Additional amino acid substitutions were identified outside the QRDR of the topoisomerase genes (Table 3), but their role in fluoroquinolone resistance was not determined. The isolate MRSP 8472 has only one amino acid substitution in *grlA* gene and remained susceptible to ciprofloxacin. No mutations were found in the *gyrB* and *grlB* genes in any of the isolates. All the isolates were susceptible to chloramphenicol, rifampicin, nitrofurantoin and linezolid. All the MRSP strains in the present study were resistant to more than three antimicrobial classes and therefore were classified as multidrug-resistant (MDR) [13].

**Table 3:**
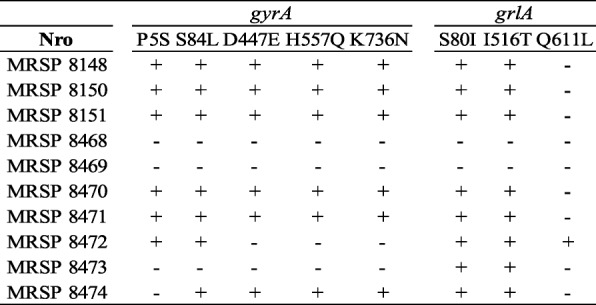
Amino acid substitutions in the topoisomerase genes of the 10 MRSP strains

### Differential levels of PBP2a expression in MRSP correlates with their β-lactam resistance

**Fig. 1:**
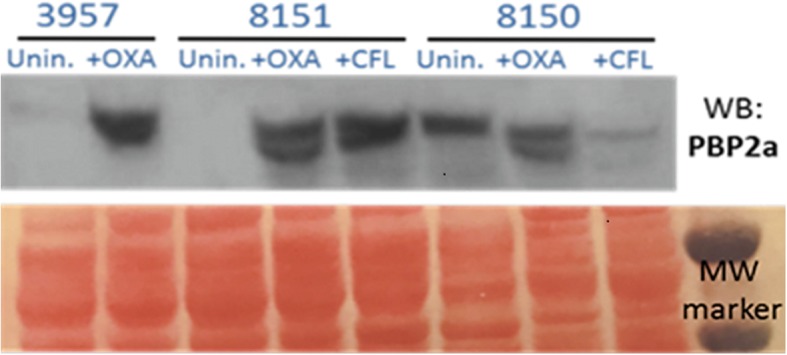
Western blot analysis of PBP2a protein in lysates of MRSP strains grown without and with subinhibitory concentrations of oxacillin (OXA) and cephalexin (CFL) at 0.5 μg/ml. Lower image correspond to Ponceau staining used as loading control. Oxacillin (OXA), cephalexin (CFL), uninduced (Unin)

Resistance to methicillin in *S. pseudintermedius*, as well as in *S. aureus* is due to the presence of the *mecA* gene, which encodes penicillin-binding protein 2a (PBP2a); this protein shows reduced affinity for all β-lactam antimicrobials. *S. aureus* strains that have the *mecA* gene incorporated in the genetic element SCC*mec* are considered to be resistant to all β-lactam antimicrobials. Analysis of PBP2a by Western blot was performed by using specific anti-PBP2a antibodies in protein lysates of MRSP strains expressing either high level of oxacillin resistance (MRSP 8150, oxacillin MIC > 256 mg/L) or low (MRSP 8151, oxacillin MIC 2 mg/L) grown without and with sub-inhibitory concentrations of oxacillin and cephalexin at 0.5μg/ml. As shown in Fig. 1, while PBP2a was inducible in strains expressing low levels of resistance (i.e MRSP 8151), it appeared to be constitutively expressed in MRSP 8150, suggesting different regulatory mechanisms in MRSP. Furthermore, the strain MRSP 8150 has both *blaI*/*blaR1* and *mecI*/*mecR1* genes unlike MRSP 8151 that only has *blaI*/*blaR1*. These results may indicate that β-lactam expression in MRSP strains could be linked to *mecA* differential regulation.

### Characterization of the SCC*mec* element in MRSP strains

**Fig. 2:**
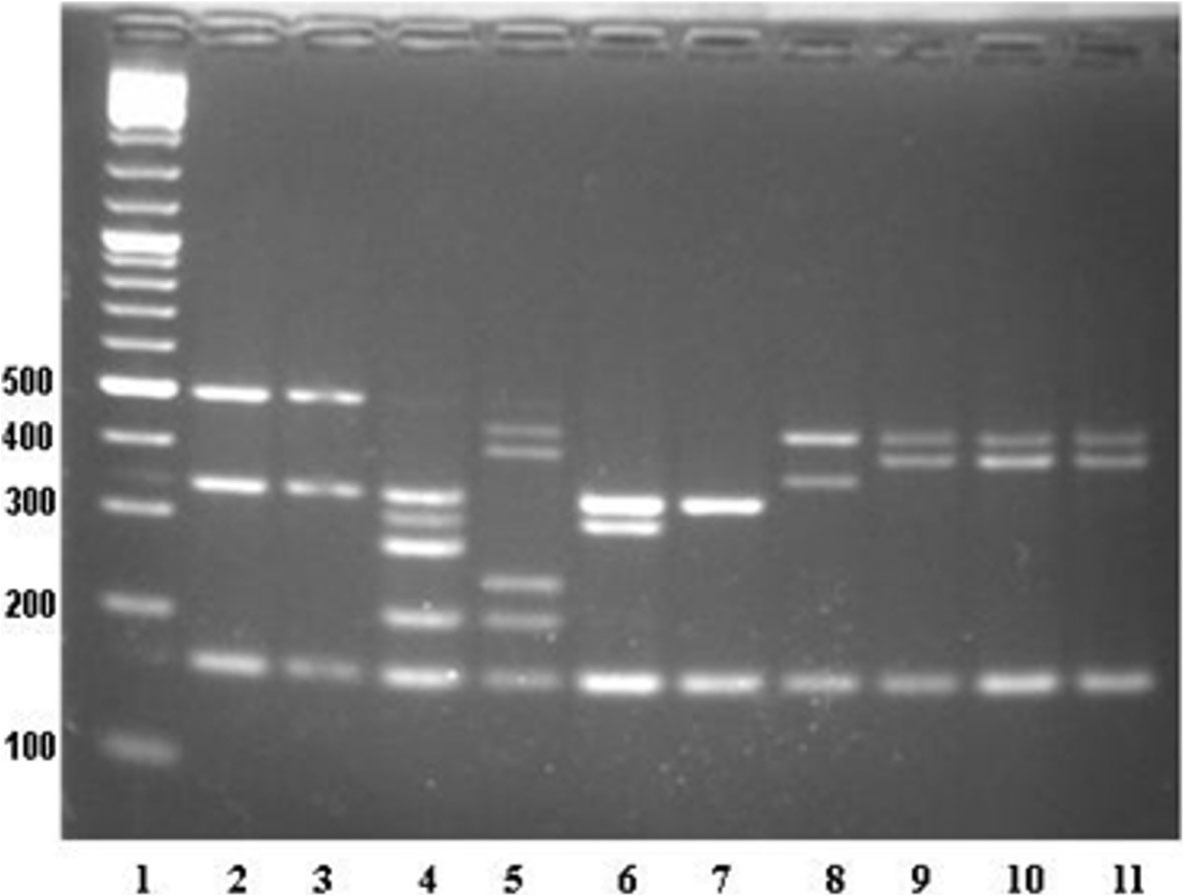
SCC*mec* characterization of the MRSP strains by multiplex PCR. Lane 1: MW marker 100 bp; lane 2: SCC*mec* I; lane 3: SCC*mec* IA; lane 4: SCC*mec* II; lane 5: SCC*mec* III; lane 6: SCC*mec* IV; lane 7: SCC*mec* VI; lane 8: SCC*mec* V; lane 9: MRSP 8148; lane 10: MRSP 8150; lane 11: MRSP 8151

Characterization of the SCC*mec* cassette was performed by multiplex PCR showing that all the isolates displayed the same pattern of bands, but different from the control isolates SCC*mec* I to VI. The pattern shared two bands with the SCC*mec* type V element corresponding to *mec*A and *ccr* complex, but differed in the band corresponding to J1 region suggesting that it could be a variant of SCC*mec* V (Fig. 2).

**Fig. 3:**
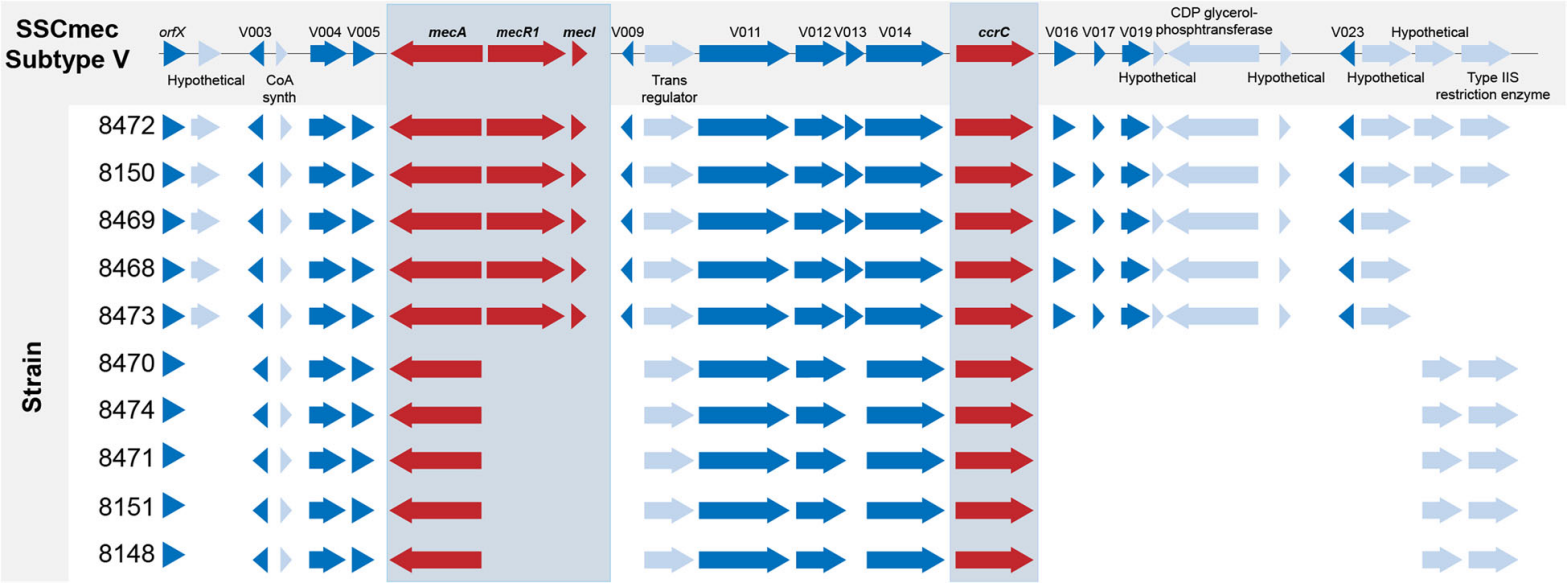
Comparison of the SCC*mec* region of the *S. aureus* TSGH17 (SCC*mec* V) with the ten isolates of our study. Five of the genomes showed high homology with the SCC*mec* V compared to the reference genome (TSGH17). The other five genomes have similar *ccr* complex and *mecA* and *ccrC* genes, but were missing many of the other genes associated with SCC*mec* V, including *mecR1* and *mecI*

Each of the strain genomes was submitted to SCC*mec*Finder [14], an in silico web-based bioinformatic tool that identifies and types SCC*mec* elements. Using this, the SCC*mec* type V (5C2 and 5) / SCC*mec* type Vb (5C2 and 5) was identified in five of the genomes, indicating that there was significant homology with *S. aureus* AB512767.1 (TSGH17), which was used as a reference. Further BLAST analysis between all the genes annotated in AB512767.1 showed that all the SCC*mec* V genes found in the cassette were present on a single contig in MRSP 8472, while the other isolates that had the genes on more than one contig. The fastq files from nine isolates were mapped against the MRSP 8472 genome to see if any reads mapped to these genes, or to other genes known to be present in the SSC*mec* cassette. This comparison showed that five of the genomes (MRSP isolates 8150, 8468, 8469, 8472 and 8473) have *mecA*, *mecR1* and *mecI*, and the majority of the genes that are present in AB512767.1 (Fig. 3). Two of these genomes (MRSP isolates 8150 and 8472) had all of the genes present in AB512767.1 and three of them (MRSP 8468, 8469 and 8473) have *mecA*, *mecR1* and *mecI*, but were missing the last two genes on the 5′ end. The remaining five genomes (MRSP 8148, 8151, 8470, 8471 and 8474) have *mecA*, but were missing many of the other genes associated with SCC*mec* V, including *mecR1* and *mecI* (Fig. 3). These results suggest that MRSP strains harboring *mecA* are differentiated into two distinct groups.

### Population structure analysis

To identify the relatedness of the MRSP strains PFGE was performed. We found that the ten MRSP strains were differentiated in eight clonal types (A to H). Moreover, identical patterns were observed between MRSP 8468 and MRSP 8469 strains (type A) while MRSP 8470 and MRSP 8471 strains displayed type B (Additional file 1: Figure S1). Additionally, MRSP type characterization was performed by using MLST as described in Methods. We found that the MRSP strains were differentiated into six different STs.

Two of them MRSP 8150 and MRSP 8472 belong to ST339 while MRSP 8468 and MRSP 8469 were related to ST649. These ST types have been described and already included in PubMLST database. The six remaining isolates had previously undescribed allelic profiles and were assigned new sequence types by the *S. pseudintermedius* MLST database curator. MRSP 8148, MRSP 8470 and MRSP 8471 belong to ST919, MRSP 8151 to ST920, MRSP 8473 to ST921 and MRSP 8474 to ST922.

**Fig. 4:**
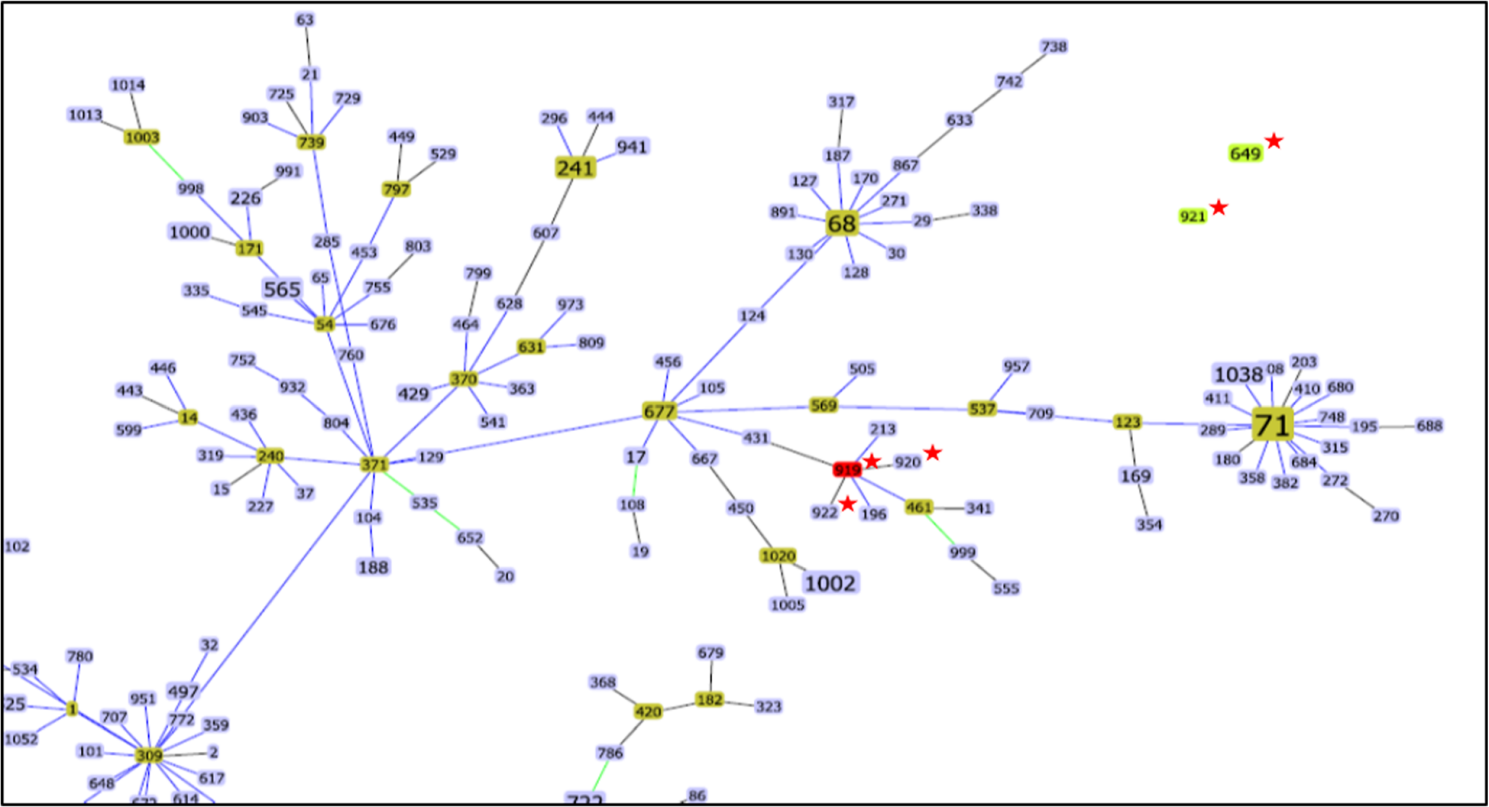
Population snapshot of MRSP. *goe*BURST analysis in which the branches are connected with a single locus variant level to show the relation of STs. Stars indicate STs from Argentina. ST339 is not shown in the figure

To determine the clonal relationship between the STs detected in this study with those found in the global Pub-MLST *S. pseudintermedius* database, all the entries available on June 2018 were clustered using the same *goe*BURST procedure (Fig. 4). The clonal complex (CC) consisted of allelic profiles with five or more allele matches, while singletons were unrelated to any other within the collection. The *goe*BURST algorithm showed that three of them (STs 919, 920 and 922) are single locus variant with each other, two (STs 649 and 921) are singletons and ST339 is part of a branch located very far from the other isolates. As shown in Fig. 4, none of the isolates in Argentina were related to ST68 or ST71. This data clearly indicates that the MRSP strains are not identical to ST68 and ST71 but evolutionary related.

### Comparative genomics and phylogenetic analyses

**Fig. 5:**
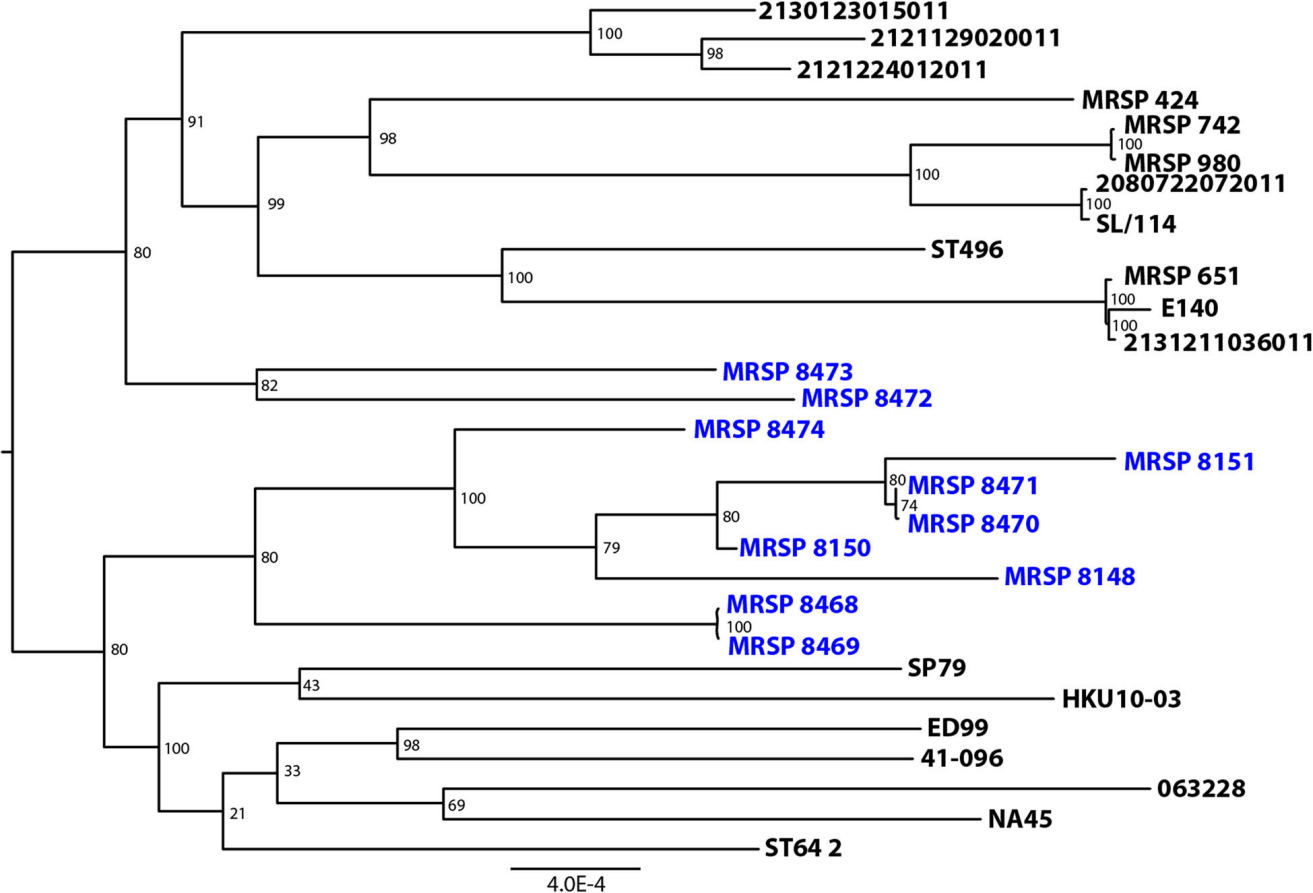
Phylogenetic tree of the ten MRSP isolates and selected representative strains. Phylogenetic tree that includes the ten MRSP isolates of the present study and a selection of nineteen previously sequenced genomes that were selected among 152 genomes and represent the phylogenetic diversity found across the species

Analysis of the MRSP genomes in PATRIC showed an average size of 2,344.838 Mb (range 2.550.634 to 2.650.119), with an average 2779 genes annotated (range 2528 to 2647). The genome composition of MRSP was found similar between the different genomes analyzed (Table 4). A phylogenetic tree that included 152 *S. pseudintermedius* previously sequenced genomes and the 10 strains in this study was generated (data not shown). Nineteen strains from the representative clades in this tree were selected for a new tree that included the 10 genomes (Table 5 and Fig. 5). The phylogenetic analysis shows that the 10 genomes in this study were not monophyletic, with MRSP 8472 and MRSP 8473 separated from the other eight isolates (Fig. 5) that clustered together.

**Table 4:** Genomic characteristics of the 10 MRSP strains

Strain	Genome ID	Contigs	Genome length (bp)	GC content (%)	CDS number
MRSP 8148	283.734.711	1	2,617,399	37.54	2528
MRSP 8150	283.734.761	153	2,650,119	37.21	2647
MRSP 8151	283.734.762	171	2,609,428	37.36	2630
MRSP 8468	283.734.763	126	2,584,184	37.32	2533
MRSP 8469	283.734.764	129	2,584,234	37.33	2540
MRSP 8470	283.734.765	142	2,564,903	37.37	2537
MRSP 8471	283.734.766	142	2,550,634	37.42	2544
MRSP 8472	283.734.767	165	2,604,960	37.24	2604
MRSP 8473	283.734.768	164	2,612,019	37.27	2598
MRSP 8474	283.734.769	168	2,621,592	37.41	2636

**Table 5:** Genomes used for phylogenetic analysis in addition to the new isolates from this study

Strain	GenBank Accession	Genome Length (bp)	Contigs	Country
E140	ANOI01000000	2769458	1	Denmark
ED99	CP002478	984892	1	United Kingdom
HKU10–03	CP002439	2617381	1	Hong Kong
063228	CP015626	2766566	1	United States
2080722072011	PEOJ01000000	2571729	101	Netherlands
2121129020011	PEPR01000000	2576622	63	Netherlands
2121224012011	PEPS01000000	2650237	98	Netherlands
2130123015011	PEPV01000000	2624816	61	Netherlands
2131211036011	PEQD01000000	2720206	100	Netherlands
41–096	MPKZ01000000	2530792	31	United States
MRSP 651	PHIB01000000	2695563	37	United States
MRSP 742	PHHY01000000	2637262	38	United States
MRSP 424	PRDQ01000000	2660157	43	United States
MRSP 980	PRDR01000000	2634738	45	United States
NA45	CP016072	2841212	1	United States
SL/114	MQND01000000	2590176	51	Sri Lanka
SP79	AP019372	2509706	1	Japan
ST496 1	QEJK01000000	2746304	226	Australia
ST64 2	QEJT01000000	2617379	347	Australia

## Discussion

*S. pseudintermedius* is a canine and feline commensal and opportunistic pathogen, analogous to *S. aureus* in humans. MRSP has recently emerged in small animals worldwide and represents a serious threat to animal health due to its characteristic multidrug resistance phenotype [15].

In this study we compared the resistance phenotype presented by ten clinical strains of MRSP with the genotypic analysis including WGS data. The cefoxitin disk test, which has been shown in several studies to be the most reliable predictor of the presence of *mecA* in both *S. aureus* and coagulase-negative *Staphylococcus*, does not identify MRSP isolates of human and veterinary origins [9, 10, 16] and would therefore not have been appropriate for the isolates in this study. Although all the isolates included in the present study were resistant to oxacillin using the current CLSI breakpoints, they could be separated into two groups, with either high or low oxacillin MICs resistance levels. Western blot analyses of PBP2a included the strains MRSP 8150 and MRSP 8151. MRSP 8150 demonstrated a high level of resistance, which was consistent with constitutive membrane levels of PBP2. The levels of PBP2a in MRSP 8151 were only inducible after exposure to oxacillin or cephalexin. The differential expression of β-lactam resistance has been observed in MRSA [17]. In previous studies, we have demonstrated that PBP2a can be co-regulated by both *mecI* and *blaI* regulators [17]. The observation that *blaI*/*blaR1* regulatory system, present in all the MRSP strains in this study (Table 3), led us to speculate that *blaI*/*blaR1* is indispensable to mediate *mecA* regulation in MRSP. In accordance with this observation, constitutive levels of PBP2a seen in MRSP 8150 may be due to defective function of *blaI*/*blaR1* despite the presence of *mecR1*/*mecI*, however this hypothesis awaits experimental confirmation and further studies are needed to demonstrate this finding.

In addition to resistance to β-lactams, MRSP isolates showed resistance to other antibiotics such as aminoglycosides, macrolides, lincosamides, tetracyclines, trimethoprim/sulfamethoxazole and fluoroquinolones. Based on these results, all the MRSP that showed resistance to five or six families of antibiotics, were categorized as MDR.

The genes responsible for the resistance to penicillin, tetracycline, erythromycin and trimethoprim/sulfamethoxazole (*blaZ*, *tetM*, *ermB* and *dfrG* genes, respectively) have also been found to be the predominating genes encoding for resistance in *S. pseudintermedius* from dogs in other studies [18, 19]. Resistance to aminoglycosides was associated with the adenyl nucleotidyltransferase gene *ant(6)-Ia* and the phosphotransferase *gene aph(3′)-III*. The bifunctional acetyltransferase/phosphotransferase gene *aac(6′)-Ie–aph(2′)-Ia* was present only in one gentamicin resistant strain. Comparable results were found in a similar study performed in a veterinary medical teaching hospital in Texas, where the most common aminoglycoside resistance gene found was *aph(3′)-IIIa*, followed by *aac(6′)/aph(2′′)* and *ant(4′)-Ia* genes [20]. Most of the resistance genes detected in *S. pseudintermedius* have also been identified in other species of staphylococci. Only the seven isolates phenotypically resistant to fluoroquinolones had mutations in topoisomerase II (*gyrA* S84 L) and IV (*grlA* S80I) simultaneously. In a previous study Descloux et al. identified numerous base pair exchanges in the genes *gyrA*, *gyrB*, *grlA* and *grlB* of *S. pseudintermedius* resistant to fluoroquinolones [21]. The same substitutions: S84 L and E88G in *gyrA* as well as S80I and D84N in *grlA*, were seen in fluorquinolone resistant MRSP isolates from Japan [22] and two others S84 L and S80R in *gyrA* identified in eight ciprofloxacin-resistant MRSP isolates from Spain [23]. Our results show excellent correlation between the resistance phenotypes and the resistance genes detected, similarly to reported recently by other authors [24].

The information gleaned by full genome sequencing of staphylococcal species allows to determine the diversity of SCC*mec* elements, the structural organization and the genetic content including genetics variants (e.g. inserts, deletions) [25]. SCC*mec* of *S. pseudintermedius* displayed some degree of homology to those of *S. aureus,* but sometimes they are untypeable using SCC*mec* typing schemes developed for *S. aureus*. The homology between SCC*mec* cassettes of different species is considered as an indication of horizontal gene transfer between isolates. SCC*mec* V is largely homologous to SCC*mec* type V (5C2&5), previously named VI or VII from *S. aureus.* Conversely, SCC*mec* II-III consists in a combination of SCC*mec* II from *S. epidermidis* and of SCC*mec* III from *S. aureus* and has lacked the cadmium resistance operon, and SCC*mec* VII-241 is a newly described element that is not related to SCC*mec* VII from *S. aureus* [26]. The SCC*mec* type III variants were found in a study carried out in a veterinary hospital from Japan, where SCC*mec* type II-III represented 85.2% of *S. pseudintermedius* isolates [27]. In our study, according to the multiplex PCR method designed by Milheirico et al for *S. aureus*, all the isolates appear to be a variant of SCC*mec* V. SCC*me*cFinder [14] could only classify five genomes as SCC*mec* type V(5C2&5)/SCC*mec* type Vb(5C2&5) due to the fragmented assemblies of the new isolates. A more detailed analysis of the genomes allowed us to observe that these five genomes not only have *mecA*, *mecR1* and *mecI*, but also have a majority of the genes that are present in the isolate used as reference of SCC*mec* V. Moreover, two of these genomes had all of the genes present in the *S. aureus* TSGH17 that was used as a reference. The other five isolates have *mecA*, but were missing many of the other genes associated with SCC*mec* V, including *mecR1* and *mecI.* Further studies are warranted to characterize the SCC*mec* element displayed by these isolates, which have the same *ccrC* recombinase but appears to be a different combination of genes than those described up to now. Although we observed differences in the SCC*mec* elements between the isolates, the differences in oxacillin MICs seem not to be associated to different SCC*mec* types, as has been recently described [8, 28].

The dissemination of MRSP isolates tended to be associated with a limited number of clones, unlike methicillin susceptible *S. pseudintermedius* isolates that presented great genetic diversity [29], similarly to the situation observed in human *S. aureus.* ST68 clone SCC*mec* V and ST71 SCC*mec* II-III are the dominant clones that have spread in North America since 2003–04 and in Europe since 2005–06, respectively, but now have a global distribution [30]. A study performed in Brazil was the first in South America to have detected the European clone ST71 of MRSP colonizing companion animals [31]. The isolates of our collection showed significant genetic variation between the population manifested by eight clonal types differentiated by PFGE and six sequence types (STs) by MLST (339, 649, 919, 920, 921 and 922), including four new STs that were genetically distinct from the previous STs in other geographic regions. The analysis by *goe*BURST of our isolates showed that they were not related to ST68 or ST71. However, ST68 and ST919 are double locus variant from ST677, which could indicate that our isolates are evolutionarily closer to ST68 than ST71. Notably the diversity of STs shown by our MRSP isolates indicates high clonal diversity in our country. We found that both internationally reported as well as previously unreported MRSP STs are present in Argentina. Giving that the clones ST919, ST920, ST921 and ST922 had not been previously reported, it is likely that they represent locally evolved clones.

## Conclusions

In summary, this is the first report addressing the phenotypic and genotypic characterization of canine MRSP isolated in Argentina between 2008 and 2011. The ability of MRSP to acquire and maintain resistance genes, and its propensity for horizontal transfer of resistance determinants have shown to represent a potential threat on both the veterinary and Public Health settings.

## Methods

### Bacterial strains and identification

Clinical samples were collected from the infected dogs at three different veterinary hospitals in Buenos Aires, Argentina between 2008 and 2011. Three strains (MRSP 8148, MRSP 8150 and MRSP 8151) were the only methicillin resistant detected in a previously studied strain collection of 28 *S. pseudintermedius* [11]. The other seven strains were recovered in two laboratories from Buenos Aires city during 2011 and fully characterized at the Antimicrobial Division, INEI-ANLIS “Dr. Carlos G. Malbrán”, Regional Reference Laboratory on Antimicrobial Resistance, Buenos Aires, Argentina The ten MRSP isolates included in the present study were isolated from infections in different body sites (Table 1). Species identification was performed by conventional biochemical tests and confirmed by mass spectrometry MALDI-TOF (Bruker Daltonics Microflex LT, Billerica MA, USA). The isolates were pheno- and genotypically characterized at the Antimicrobial Division, INEI-ANLIS “Dr. Carlos G. Malbrán”.

### Susceptibility testing of MRSP isolates

*S. pseudintermedius* strains were tested by disk diffusion to evaluate their antimicrobial susceptibility to the following antibiotics (disk concentration in brackets): oxacillin (1 μg), cefoxitin (30 μg), penicillin (10 units), erythromycin (15 μg), clindamycin (2 μg), tetracycline (30 μg), chloramphenicol (30 μg), rifampicin (5 μg), gentamicin (10 μg), streptomycin (10 μg), kanamycin (30 μg), trimethoprim-sulfamethoxazole (1.25/23.75 μg), nitrofurantoin (300 μg), linezolid (30 μg) and ciprofloxacin (5 μg). Oxacillin MIC was determined by agar dilution in MH agar + 2% NaCl (CLSI) with a range of antibiotic concentrations from 0.03 to 8 mg/L, and by Etest (bioMérieux, France). All antimicrobial susceptibility tests were carried out according to the CLSI guidelines [32, 33]. *S. pseudintermedius* strains were categorized as susceptible, intermediate, or resistant, when the applicable breakpoint was available in CLSI documents VET01S-3rd ed., 2015 or M100S-27th ed., 2017. Despite the lack of CLSI-approved interpretative criteria for streptomycin and kanamycin, the isolates for which the inhibition zone was 6 mm were considered as resistant. Isolates were considered as multidrug-resistant when they exhibited resistance to three or more different classes of antimicrobial agents [13].

### PBP2a analysis in MRSP strains

Western blot analysis was used to determine changes in PBP2a levels as previously described [34]. Briefly, membrane proteins (15 μg) will be extracted from MRSP strains growing with/without sub-inhibitory concentrations of oxacillin and cephalexin (CFL) at 0.5 μg/ml in MHB until mid-exponential phase; cell pellets were resuspended in 600 μl of phosphate-buffered saline (PBS), disrupted by adding glass beads and using a FastPrep cell disrupter (MP Biomedicals, Santa Ana, CA, USA); the lysate was centrifuged at 8,000×g for 10 min at 4 °C. The supernatant fraction was centrifuged for an additional 5 min at 8,000×g at 4 °C to remove the beads, and the supernatant transferred to ultracentrifuge tubes and ultracentrifuged at 45,000 rpm for 1 h/4 °C. The membrane pellet was resuspended in PBS, total membrane proteins quantified and stored at − 80 °C. Lysates were separated on 4 to 12% bis-Tris gels, blot transferred onto pure nitrocellulose blotting membranes, and after blocking (5% low-fat milk in PBS), PBP2a was probed with monoclonal anti-PBP2a antibody (Slidex MRSA detection kit; bioMérieux, France).

### Genotyping

#### *mecA* gene PCR

All the MRSP strains were tested for the presence of the *mecA* gene by PCR. PCRs were performed as previously described [35], *S. aureus* ATCC 43300 and *S. aureus* ATCC 29213 were used as positive and negative control, respectively.

#### Identification of SCC*mec* elements among MRSP strains

MRSP isolates were first screened for typical SCC*mec* elements by multiplex PCR as previously described [36]. *S. aureus* collection strains were used as control of each SSC*mec* type: COL, PER34, BK2464, USA100, ANS46, HU25, USA400, a clinical strain and HDE288 were used as positive control of SCC*mec* types I, IA, II, III, IV, V and VI respectively [36]. The genome sequence from each of the 10 new isolates was examined in the SSC*mec*Finder resource [14] to determine the SSC*mec* type. In addition, the SSC*mec* V (GenBank Id AY894416) [37] nucleotide and individual protein sequences were compared by BLAST [38] to the new genomes in PATRIC [39]. A careful examination of the region containing the SCC*mec* V genes and its flanking regions was conducted using the Proteome Comparison and Compare Region View [40] tools found in PATRIC. A broad examination for the presence or absence of the protein families that contain *mecA*, *mecR1* and *mecI* genes across all *S. pseudintermedius* genomes was conducted using PATRIC’s Protein Family Sorter [41].

As the MRSP 8472 genome had all the genes in the SSC*mec* element present on a single contig, the reads from the remaining nine genomes were mapped to that genome using PATRIC’s variation service to confirm the presence or absence of the genes in the SCC*mec* V region. Genes were considered present when reads were present that overlapped both the 5′ and 3′ ends of the genes in MRSP 8472, as well as covering more than 60% of the total length of the gene.

#### Pulsed-field gel electrophoresis (PFGE) analysis

Chromosomal DNA of the MRSP strains digested with *Smal* was analyzed by PFGE, as described previously [42]. PFGE was carried out by clamped homogeneous electric field electrophoresis with a CHEF DR III System (Bio-Rad Laboratories, Richmond, CA, USA). PFGE was performed under the following conditions: switch time, 2.0 to 20.0 s and run time, 20 h; temperature 11.3 °C, angle 120° and voltage 6 V/cm. Separated DNA fragments were stained with ethidium bromide and visualized with a UV transilluminator. Banding patterns were evaluated by visual inspection and interpreted according to Tenover criteria [43]. Isolates were considered unrelated when the PFGE patterns differed in seven or more bands, consistent with three or more independent genetic events.

### Genome sequencing

Genomic DNA was extracted by using DNeasy Blood and Tissue Kit (QIAGEN, Valencia, CA, USA) as per manufacturer’s instructions; concentration was measured by QubitTM assay (Invitrogen, Carlsbad, CA, USA). Illumina library preparation was carried out by Nextera XT DNA Library Preparation Kit (Illumina, San Diego, CA, USA). Hi-seq sequencing was carried out in our affiliated Weill Cornell University (New York, NY, USA) institution at the Epigenetics and Genomic Laboratory, using an Illumina HiSeq 2000. Assembly, annotation and analysis of genomes were done through the PATRIC software (https://www.patricbrc.org). The detection of resistance genes was carried out with PATRIC using the available ResFinder (genomicepidemilogy.org) and CARD (Comprehensive Antimicrobial Resistance Database, card.macmaster.ca) databases, the gene content were compared with the phenotype presented by them.

### Population structure analysis

Sequence types were determined using MLST software (https://bio.tools/mlst). Sequence types were assigned by comparison with the allele sequences present in the PubMLST database (http://pubmlst.org/spseudintermedius) and isolates with a novel combination of alleles were submitted to the MLST database curator Vincent Perreten (vincent.perreten@vetsuisse.vbi.unibe.ch). We determine the clonal relationships of the sequence types obtained in this study with entries in the global PubMLST *S. pseudintermedius* database. All entries available at the time of analysis were clustered using the same *goe*BURST procedure database (http://www.phyloviz.net/goeburst/).

### Phylogenetic trees

An initial tree including 152 *S. pseudintermedius* genomes, including the 10 isolates sequenced in this study, was created to select appropriate genomes to represent the phylogenetic diversity found across the species. Genomes were selected based on clusters identified in this tree, and as a result, nineteen previously sequenced genomes representing these branches were selected to be compared with the 10 Argentinian genomes.

Protein families from genes that were present as a single copy per genome were selected, and 1000 of these Global protein families (PGFams) [44] were used. Both the protein (amino acid) and gene (nucleotide) sequences were used for each of the selected genes. Protein sequences were aligned using MUSCLE [45], and the nucleotide coding gene for each was aligned using the Codon_align function of BioPython [46]. A concatenated alignment of all proteins and nucloetides were written to a phylip formatted file, and then a partitions file for RaxML [47] was generated, describing the alignment in terms of the proteins and then the first, second and third codon positions. Support values were generated using 100 rounds of the “Rapid” bootstrapping option [48] of RaxML. The resulting newick file was viewed in FigTree [49].

## Additional file

Additional file 1: **Figure S1.** PFGE of 10 MRSP strains digested with *SmaI*. Lane 1: MW marker; lane 2: MRSP 8148; lane 3: MRSP 8150; lane 4: MRSP 8151; lane 5: MRSP 8468; lane 6: MRSP 8469; lane 7: MRSP 8470; lane 8: MRSP 8471; lane 9: MRSP 8472; lane 10: MRSP 8473; lane 11: MRSP 8474. (TIF 463 kb)

## Data Availability

The datasets used and/or analysed during the current study are available from the corresponding author on reasonable request.

## Abbreviations

CC: Clonal complex
CFL: Cephalexin
CLSI: Clinical and Laboratory Standards Institute
IWG-SCC: International Working Group on the Staphylococcal Cassette Chromosome
MDR: Multidrug-resistant
MHB: Mueller-Hinton broth
MIC: Minimal inhibitory concentration
MLST: Multilocus-sequence typing
MRSA: Methicillin-resistant *S. aureus*
MRSP: Methicillin-resistant *S. pseudintermedius*
PBP: Penicillin-binding protein
PFGE: Pulsed-field gel electrophoresis
QRDR: Quinolone resistance-determining region
SCC*mec*: Staphylococcal cassette chromosome
SIG: *S. intermedius* group
ST: Sequence type
WGS: Whole-genome sequencing
WHO: World Health Organization
