# Supplementary figures and images for: Identification and molecular epidemiology of methicillin resistant Staphylococcus pseudintermedius strains isolated from canine clinical samples in Argentina
Source: BMC Vet Res. 2019 Jul 27;15:264. doi: 10.1186/s12917-019-1990-x (PMC6660709; doi:10.1186/s12917-019-1990-x)

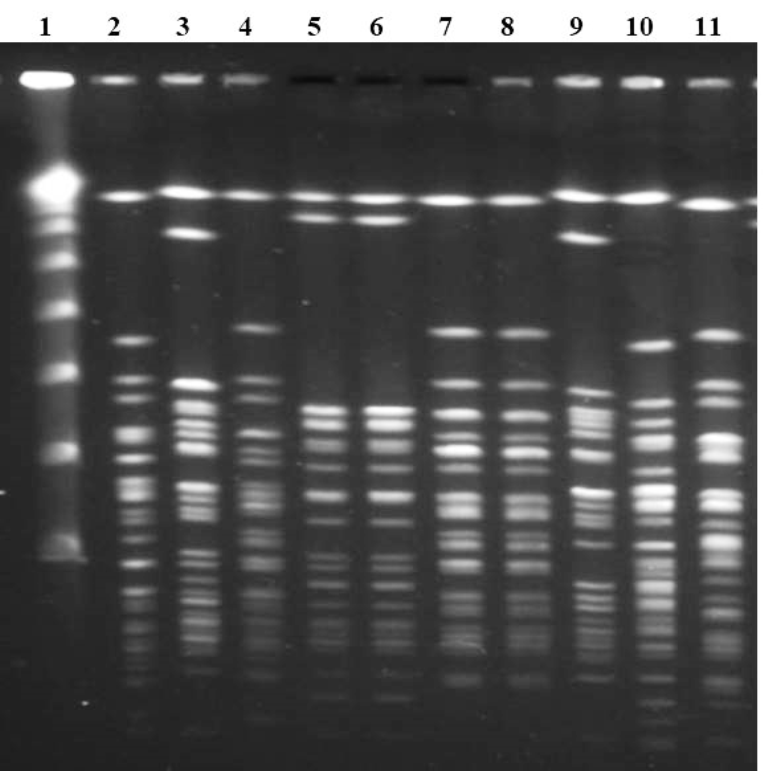

Supplement: Supplementary file 1 — Figure S1. PFGE of 10 MRSP strains digested with SmaI. Lane 1: MW marker; lane 2: MRSP 8148; lane 3: MRSP 8150; lane 4: MRSP 8151; lane 5: MRSP 8468; lane 6: MRSP 8469; lane 7: MRSP 8470; lane 8: MRSP 8471; lane 9: MRSP 8472; lane 10: MRSP 8473; lane 11: MRSP 8474. (TIF 463 kb) [file 12917_2019_1990_MOESM1_ESM.tif]
